# Adult Food Security and the Relationship with Adverse Childhood Experiences Among Residents of Appalachian North Carolina

**DOI:** 10.13023/jah.0103.03

**Published:** 2019-09-27

**Authors:** Manan Roy, Erin Bouldin, Maggie Bennett, Adam Hege

**Affiliations:** Appalachian State University; Appalachian State University; Appalachian State University; Appalachian State University

**Keywords:** Appalachia, adverse childhood experiences, health disparities, health outcomes, lifespan, social determinants of health, risk factors, food insecurity

## Abstract

**Introduction:**

The Appalachian region has worse health outcomes than the remainder of the United States. These disparities are often linked to the underlying social and environmental determinants of health. Adverse childhood experiences (ACEs) are associated with poor health outcomes across the lifespan and have a significant impact on future social determinants as an adult, including food security status.

**Purpose:**

To explore the relationships between ACEs and food security among adults in the Appalachian counties of North Carolina and make comparisons with the rest of the state.

**Methods:**

Researchers used North Carolina’s 2012 Behavioral Risk Factor Surveillance System data; namely, the ACEs optional module which includes 11 items related to experiences respondents had before the age of 18 and a single item from the Social Context optional module to classify food security status. The sample was divided into three age categories (18–44, 45–64, and 65 and older) for statistical comparisons as well as by the indicator for Appalachian county. Using Stata 15, weighted logistic regression was utilized for examining relationships between variables.

**Results:**

ACEs were a statistically significant predictor of food insecurity across all respondents; each additional ACE was associated with a 13–21% increase in the odds of food insecurity, depending on age group. However, living in an Appalachian county was only a predictor for those age 45–64.

**Implications:**

These findings highlight the long-term effects of childhood experiences on food security generally, and in Appalachia particularly for middle-aged adults. Reducing ACEs could reduce food insecurity and improve health in the region.

## INTRODUCTION

Recent reports highlight health disparities across the Appalachian region, which includes 13 states and more than 400 counties. Many of these health disparities are rooted in social determinants of health, including socioeconomic status and opportunity, healthcare access, transportation and the built environment, and food security.^1^ In addition, Appalachia is predominantly rural and has a unique social and cultural context that predisposes many areas to face challenges across generations.^2^

Food insecurity, which includes experiences ranging from anxiety about purchasing food of adequate quality or in sufficient quantity to reducing food intake, is linked to numerous adverse health outcomes.^3^,^4^ The growing prevalence of food insecurity in the U.S. is a critical public health issue and is of great concern to policymakers and government officials. National figures showed 12.9% of U.S. households, or approximately 41 million citizens, were food insecure at least some time during 2016.^3^

Adverse childhood experiences (ACEs) include abuse—physical, sexual, and emotional—and household dysfunctions, including substance abuse, separation or divorce, violence between adults, mental illness, and incarceration.^5^,^6^ ACEs are associated with numerous poor health and social outcomes across the lifespan, including poor physical and mental health and mortality.^5^,^7^

Existing research has established the importance of various social, economic, and behavioral factors that can explain household food insecurity. However, as Temple^8^ notes, stressful life events or stressors such as mental illness, not being able to get a job, or death of a family member or close friend, have received scant attention in the empirical literature on food insecurity. Using nationally representative data from Australia, Temple showed that exposure to 18 discrete stressors significantly increased the probability of experiencing food insecurity, while adjusting for the more established correlates of food insecurity, like lower education level, being unmarried, or renting a home. Here in the U.S., Chilton and colleagues^9^ found that exposure to ACEs was associated with experiencing food insecurity later in adulthood. Further, using the Midlands Family Study, researchers reported that low-income families with inadequate social support who adjust to adverse life events have a higher probability of child hunger.^10^

## PURPOSE

In this light, the current study sought to examine the relationship between ACEs and food insecurity among North Carolina (NC) residents. Four main factors led to studying this relationship. First, the study addresses an important omission in the literature on Appalachian outcomes: population-based long-term effects of ACEs on stress-related food insecurity accounting for residence in an Appalachian county. Second, the study focused on Appalachian NC since NC experienced a food insecurity rate of 14.4% in 2015–2017 while the national average food insecurity rate was 12.3%.^3^ Third, the *Appalachian Diseases of Despair* report commissioned by the Appalachian Regional Commission (ARC) finds that in Appalachian NC, the diseases of despair mortality rate was 45% higher than in the non-Appalachian portions in 2015.^11^,^12^ Finally, although not statistically different from the national prevalence rate, the prevalence of 3–8 ACEs among NC residents exceeded the corresponding national prevalence rate in 2016.^11^

## METHODS

### Data

Data come from the 2012 Behavioral Risk Factor Surveillance System (BRFSS) in NC. BRFSS is a landline and cell phone survey of community-dwelling adults aged 18 and older designed to represent the state’s population in terms of age, gender, race/ethnicity, home ownership, and telephone type. The primary independent and dependent variables in this study, ACEs and stress-related food insecurity, were each included in the optional modules of the NC BRFSS only in 2012.

### Measures

The ACEs optional module includes 11 items that relate to experiences respondents had before age 18. Specifically, respondents were asked whether or not they experienced any of the following:

- (1) lived with someone who was depressed, mentally ill, or suicidal;
- (2) lived with anyone who was a problem drinker or alcoholic;
- (3) lived with anyone who used illegal street drugs or who abused prescription medications;
- (4) lived with anyone who served time or was sentenced to serve time in a prison, jail, or other correctional facility; or
- (5) parents separated or divorced.

In addition, respondents were asked how often (never, once, or more than once) before age 18:

- (6) parents or adults in their home ever slapped, hit, kicked, punched, or beat each other up;
- (7) parent or adult in their home ever hit, beat, kicked, or physically hurt them in any way (except spanking);
- (8) parent or adult in their home ever swore at them, insulted them, or put them down;
- (9) anyone at least 5 years older than them or an adult, ever touched them sexually;
- (10) anyone at least 5 years older than them or an adult tried to make them touch them sexually;
- (11) anyone at least 5 years older than them or an adult forced them to have sex.

Respondents were classified as having experienced an ACE if they had ever experienced it (i.e., “ever” or “once” or “more than once,” depending on the item).

Four measures of ACEs were created based on an earlier study by Ford and colleagues^13^:

1. a continuous ACE score by summing the number of “yes” and “ever” responses, with a possible range of 0–11 ACEs; and three dichotomous subscales:
2. household dysfunction,
3. emotional or physical abuse, and
4. sexual abuse.

Household dysfunction included items 1–5. Physical or emotional abuse included items 6–8. Sexual abuse included items 9–11.

The BRFSS includes a single question in the Social Context module to classify respondents as experiencing food-related stress (food insecurity): “How often in the past 12 months would you say you were worried or stressed about having enough money to buy nutritious meals?” Respondents who said they were always, usually, or sometimes worried or stressed were classified as experiencing food-related stress (food insecurity) and those who said they rarely or never were worried or stressed were classified as not experiencing food insecurity. Previous studies^14^,^15^ have validated the high correlation between the single measure of food-related stress or food insecurity in the BRFSS with the U.S. Department of Agriculture’s Current Population Survey Food Security Supplement. Note the BRFSS measure may reflect marginal food security rather than more severe food insecurity.^16^ For consistency, “food insecurity” is used instead of food-related stress.

Respondents were also classified as living within or outside Appalachia based on their county of residence. Counties were considered to be part of Appalachia (n=29) based on the ARC classification. The NC BRFSS coordinator created the Appalachia indicator variable since the public BRFSS files no longer include county.

Respondents self-reported a variety of socioeconomic and demographic characteristics, including age, gender, highest level of education completed, race and ethnicity, current employment status, marital status, the number of children under 18 living in their household, annual household income, home ownership status, and whether they ever served on active duty or in the reserves.

### Statistical Analysis

Existing research^4^ finds that younger people and households with children are more likely to be food insecure. Further, mental health problems often emerge in early adulthood such that it is important to study the link between ACEs and food insecurity for that age group.^17^ Adults aged ≥45 years are most affected by the ongoing crises of “diseases of despair” in most Appalachian states. Looking at this age group separately may signal a pathway between ACEs and the “diseases of despair” for this age group.^18^,^19^ North Carolina has a higher than average rate of opioid deaths: the rate was 19.8 per 100,000 in the state in 2017 compared to 14.6 per 100,000 nationally.^20^ The majority of these deaths in North Carolina occurred among younger and middle-aged adults; in 2017, 30% of opioid-related deaths in the state were among those aged 25–34, 25% were among people aged 35–44, and 21% were among adults aged 45–54.^21^ Older adults also experience food insecurity, but in general food insecurity declines with age.^22^ Finally, early mortality associated with ACEs may result in differential associations between food insecurity and ACEs by age group.

Accordingly, the sample was divided into three age categories: 18–44, 45–64, and ≥ 65. T-tests were used to evaluate whether the prevalence of food insecurity, ACE measures, and other demographic characteristics differed significantly across the three age categories. For each age category, logistic regression models were estimated to assess the relationship between each of the ACE measures and food insecurity, controlling for the social, economic, and demographic characteristics listed above as well as an indicator for residing in an Appalachian county. For each age group, four regression models were estimated, one for each measure of ACE. For each ACE measure, whether living in Appalachia had a differential effect on food insecurity was tested. All analyses were weighted with BRFSS survey weights to represent the state’s population. The data were analyzed using Stata 15.

## RESULTS

There were 11,770 respondents included in the study: 3661 aged 18–44; 4424 aged 45–64; and 3685 aged ≥65. The mean ACE score decreased with age (Table 1; all tables are provided under Additional Files). Household dysfunction was the most commonly reported type of ACE across all age groups and sexual abuse was least common; the prevalence of each ACE category generally decreased with age. Respondents aged ≥65 were more likely to be female, white, and veterans than respondents aged 18–44. They also tended to have less education and were less likely to have children in the household and to rent their home than those aged 18–44. Younger respondents were less commonly residents of an Appalachian county than older respondents; this is not surprising as recent data have shown that rural counties, such as found in Appalachia, have higher rates of residents aged ≥65.^23^ Echoing other studies,^4^ younger respondents had the highest rate of food insecurity (30%) compared to 25% among those aged 45–64, and only 13.2% for the elderly.

Table 2 (provided under Additional Files) shows the continuous ACE score increased the likelihood of food insecurity for all age groups (p<0.01), after adjusting for covariates. Each additional ACE was associated with a 13%–21% increase in the odds of food insecurity as an adult. No independent effect of living in Appalachia was found in any of the age groups nor were there any differential effect of ACE by Appalachian county. The estimated odds for the differential effect of ACEs by Appalachian county (for Tables 2–5) are available on request.

Table 3 (provided under Additional Files) shows that experiencing household dysfunction as a child increased the odds of food insecurity in adulthood by 44%–54% only in the younger and middle age groups (p<0.01). Similar to the findings in Table 2, there was no independent effect of living in Appalachia in any of the age groups nor were there any differential effect of experiencing household dysfunction in childhood by Appalachian county.

Unlike the household dysfunction subscale of ACE, Table 4 (provided under Additional Files) shows that experiencing any physical and emotional abuse in childhood increased the odds of food insecurity for all age groups: by 56%–90% for all age groups. Moreover, for the 45–64 age group, residing in an Appalachian county increased the odds of food insecurity (p<0.05). However, there was no differential effect of physical and emotional abuse by Appalachian county for any of the age groups.

Finally, as shown in Table 5 (provided under Additional Files), the odds of food insecurity increased by 65%–108% if younger and middle-aged respondents experienced any sexual abuse in childhood (p<0.01). Only for the 45–64 age group, residing in an Appalachian county increased the odds of food insecurity (p<0.05). However, there was no differential effect of experiencing sexual abuse in childhood by Appalachian county.

## IMPLICATIONS

The results corroborate earlier findings that ACEs increase the likelihood of being food insecure in adulthood, especially for the groups aged 18–44 and 45–64 years. The significant effect of ACEs on food insecurity in young adulthood could be due to emergence of mental health problems such as depression and substance abuse during this period.^17^

For those aged 45–64, physical and emotional abuse and sexual abuse in childhood had the greatest odds of predicting adult food insecurity. The Opioid Risk Tool, developed by Lynn R Webster, MD, also lists personal pre-adolescent sexual abuse as one of the risk factors for opioid abuse. Although accounting for opioid abuse is beyond the scope of this paper, these independent findings warrant further research about the connections between ACEs, adult food insecurity, and other social determinants of health, and adult opioid use in other areas of the Appalachian region.

The lack of a consistent relationship between ACEs and food insecurity for the elderly could, among others, be explained by potential recall error as well as unwillingness to report or discuss such events. Also, in general, residence in one of the Appalachian counties was not associated with food insecurity after adjusting for other covariates. However, among people in the 45–64 age range, living in an Appalachian county independently increased the odds of food insecurity when controlling for physical/emotional abuse or sexual abuse in childhood. Here too, there were no differential effects by Appalachian county of residence.

The first contribution of the study is assessing the population-based long-term effect of ACEs and adult food insecurity in a mostly rural Appalachian state such as NC. The second contribution is using different definitions of ACEs—the total number of ACEs as well as individual subscales depicting household dysfunction, physical/emotional abuse, and sexual abuse. This distinction throws light on the type of ACE driving the relationship. The other contribution is the focus on different age categories since ACEs have differential effects over the course of a lifetime. Specifically, the study found that physical and emotional abuse in childhood had the greatest odds of predicting adult food insecurity for adults who were at least aged 45. Sexual abuse in childhood was most strongly associated with adult food insecurity for the youngest age group. It is possible that older respondents have had more life experiences and developed coping mechanisms whereby physical and emotional abuse dominate adult life experiences. Interestingly, although household dysfunction was the most commonly reported type of ACE across all age groups, it was not the strongest predictor of adult food insecurity for any age group. These results suggest that individual trauma (physical/emotional abuse or sexual abuse) in childhood may have stronger long-term effects than a dysfunctional environment.

The principal limitation of the study is that a causal relationship between ACEs and food insecurity cannot be estimated, especially in the presence of potential recall error or misreporting ACEs.^24^,^25^ In addition, only a single item was used to assess food insecurity, which may reflect marginal food security rather than more severe food insecurity.

The findings from this study illustrate life course impacts of the social determinants of health, in the form of ACEs and adult food insecurity, and the need for comprehensive and upstream approaches for the challenges facing Appalachia. While the ACE definitions in the study include only household dysfunction, physical and emotional, and sexual abuse, future research should ideally include a broader definition of ACEs such as additional peer- and community-related factors that also count as harmful childhood adversities.^6^

For future generations, it is vital that researchers, program developers, and policymakers translate the growing evidence into action by designing preventative and family-based programs which provide adequate resources and support, such as the Building Community Resilience Model,^26^ that systematically address social determinants of health early in life before adverse health effects occur as well as help develop resilience in the face of adversities.^10^

**SUMMARY BOX**

**What is already known about the topic?** Food insecurity and adverse childhood experiences (ACEs) are major public health issues across the United States that are linked to other social determinants of health and poor health outcomes across the lifespan.

**What is added by this report?** This study complements the limited research on the relationship between food insecurity and ACEs in Appalachia. The study uses different definitions of ACEs and focuses on different age categories since ACEs have differential effects over the course of one’s life.

**What are the implications for public health, practice, policy, and research?** Future research should include a broader definition of ACEs such as additional peer- and community-related factors that also count as harmful childhood adversities. Researchers, program developers, and policymakers should focus on designing preventative and family-based programs which provide adequate resources and support to systematically address social determinants of health early in life before adverse health effects occur as well as help develop resilience in the face of adversities.

**Table 1:** Descriptive statistics: Food insecurity, ACEs, and other covariates, by age group

	(1) Respondents aged 18–44			(2) Respondents aged 45–64			(3) Respondents aged ≥65		
Variables	N	Mean or %	SD or 95% CI	N	Mean or %	SD or 95% CI	N	Mean or %	SD or 95% CI
**Outcome**									
Food insecurity	3324	30.0	[28.5–31.6]	4230	25.0**	[24.3–26.9]	3493	13.2**	[12.1–14.4]
**Adverse Childhood Experiences (ACE)**									
Continuous ACE score	3055	1.9	2.3	3864	1.6^***^	2.0	3212	0.8**	1.4
Any household dysfunction	3199	55.8	[53.8–57.2]	4059	46.4**	[44.7–47.7]	3380	28.3**	[26.8–29.8]
Any physical and emotional abuse	3170	41.4	[40.2–43.6]	4006	38.0**	[36.4–39.4]	3300	22.6**	[21.2–24.0]
Any sexual abuse	3202	12.6	[11.5–13.8]	4072	12.0	[11.9–14.0]	3377	6.4**	[5.6–7.3]
**Covariates**									
*Demographics*									
Age (reported age in years)	3661	32.5	7.5	4424	54.3**	5.6	3685	74.2**	7.2
Men	3661	43.0	[41.4–44.6]	4424	39.6**	[38.2–41.1]	3685	34.8**	[33.3–36.4]
White non-Hispanic	3661	56.7	[55.1–58.3]	4424	69.0**	[67.6–70.4]	3685	79.4**	[78.1–80.7]
Black non-Hispanic	3661	19.9	[18.7–21.3]	4424	19.5	[18.4–20.7]	3685	13.3**	[12.2–14.4]
Any race Hispanic	3661	13.4	[12.4–14.6]	4424	3.3**	[2.82–3.88]	3685	1.05**	[0.7–1.4]
Other/Multiple race non-Hispanic	3661	9.3	[8.4–10.3]	4424	7.6**	[6.8–8.4]	3685	5.0**	[4.3–5.8]
Married or partnered	3649	52.9	[51.3–54.6]	4412	58.2**	[56.8–59.7]	3677	46.0**	[44.4–47.6]
Veteran	3661	7.4	[6.6–8.3]	4424	11.9**	[10.9–12.8]	3685	20.0**	[18.7–21.3]
*Education*									
Less than HS	3661	11.1	[10.2–12.2]	4424	10.1	[9.2–10.9]	3685	17.4**	[16.2–18.6]
High school degree or equivalent	3661	26.6	[25.2–28.1]	4424	28.9*	[27.6–30.3]	3685	31.6**	[30.2–33.2]
Some college	3661	29.2	[27.8–30.7]	4424	27.4	[26.0–28.7]	3685	24.3**	[22.9–25.7]
College degree or higher	3661	32.8	[31.3–34.3]	4424	33.4	[32.0–34.8]	3685	26.2**	[24.8–27.6]
Missing education	3661	0.001	[0.000–0.002]	4424	0.002*	[0.001–0.004]	3685	0.004*	[0.002–0.006]
*Economic factors*									
Currently employed	3641	68.7	[67.2–70.3]	4410	56.4**	[54.9–57.9]	3681	11.6**	[10.5–12.6]
Annual household income <$15,000	3661	12.3	[11.3–13.4]	4424	12.4	[11.4–13.4]	3685	12.0	[10.9–13.1]
Annual household income $15,000 – 24,999	3661	18.8	[17.5–20.0]	4424	14.4**	[13.3–15.4]	3685	0.19	[18.3–20.8]
Annual household income $25,000 – 49,999	3661	22.5	[21.2–23.9]	4424	22.9	[21.7–24.2]	3685	23.3	[21.9–24.7]
Annual household income $50,000 – 74,999	3661	12.6	[11.6–13.7]	4424	13.3	[12.2–14.3]	3685	9.0**	[8.1–9.9]
Annual household income ≥ $75,000	3661	20.2	[18.9–21.5]	4424	23.7**	[22.5–25.0]	3685	10.7**	[9.7–11.7]
*Household characteristics*									
Any children in household	3649	57.5	[55.9–59.1]	4419	19.9**	[18.7–21.1]	3681	2.6**	[2.1–3.1]
Own home	3661	49.7	[48.1–51.3]	4424	77.0**	[75.7–78.2]	3685	83.2**	[82.1–84.5]
Rent	3661	40.3	[38.7–41.9]	4424	18.4**	[17.3–19.6]	3685	12.2**	[11.2–13.3]
*Regional characteristics*									
Live in an Appalachian County	3661	15.9	[14.8–17.2]	4424	19.1**	[17.9–20.2]	3685	22.1**	[20.8–23.5]

* Notes: N denotes the number of observations. SD denotes standard deviation. 95%CI denotes 95% confidence interval. All analyses are weighted by BRFSS survey weights. The ACE score is a sum of 11 categories of ACEs. The ACE subscale of household dysfunction represents whether, as a child, the respondent lived with divorced/separated parent, or with someone who used illegal drugs, had drinking problems, or was incarcerated. The ACE subscale of physical or emotional abuse represents whether the respondent was exposed to swearing, punching, or parents beating each other in childhood. The ACE subscale of sexual abuse represents whether the respondent was touched sexually by someone, or forced to touch someone sexually, or forced to have sex with an adult in childhood. Significant difference in means or percentages relative to the category aged 18–44 years denoted by

* p<0.05,

** p<0.01.

**Table 2:** Association between food insecurity and continuous ACE score, by age group

	Odds of food insecurity by age group		
Independent variables↓	(1) Respondents aged 18–44	(2) Respondents aged 45–64	(3) Respondents aged ≥65
Continuous ACE score	1.158***	1.206***	1.134***
	(0.0265)	(0.0333)	(0.0514)
Appalachian County	1.108	1.252	1.154
	(0.153)	(0.174)	(0.201)
Female	1.157	1.546***	1.301
	(0.136)	(0.196)	(0.261)
High school degree or equivalent	0.831	0.729*	0.568***
	(0.147)	(0.130)	(0.107)
Some college	0.947	0.772	0.637**
	(0.175)	(0.147)	(0.132)
College degree or higher	0.676*	0.448***	0.731
	(0.143)	(0.0917)	(0.179)
Black, non-Hispanic	0.733**	0.803	1.484*
	(0.106)	(0.120)	(0.315)
Any race, Hispanic	1.243	1.078	1.382
	(0.207)	(0.298)	(0.891)
Other/Multiple race, non-Hispanic	0.731	1.529*	1.733*
	(0.160)	(0.348)	(0.533)
Age in years	1.182**	1.292	0.952
	(0.0858)	(0.279)	(0.221)
Squared age	0.998*	0.997	1.000
	(0.00112)	(0.00196)	(0.00153)
Married or partnered	1.030	0.912	1.073
	(0.134)	(0.114)	(0.190)
Currently employed	0.793*	0.603***	1.433
	(0.0957)	(0.0721)	(0.350)
Any children in household	0.982	1.706***	2.111**
	(0.112)	(0.261)	(0.787)
Annual household income <$15,000	1.641**	3.520***	3.561***
	(0.324)	(0.746)	(0.840)
Annual household income $15,000 – 24,999	1.154	2.537***	2.419***
	(0.208)	(0.463)	(0.502)
Annual household income $25,000 – 49,999	0.755	0.975	0.598**
	(0.139)	(0.168)	(0.134)
Annual household income $50,000 – 74,999	0.345***	0.472***	0.222***
	(0.0839)	(0.104)	(0.105)
Annual household income ≥ $75,000	0.227***	0.123***	0.0625***
	(0.0658)	(0.0333)	(0.0412)
Own home	1.250	0.635*	0.849
	(0.269)	(0.151)	(0.259)
Rent	1.736***	0.921	1.823*
	(0.337)	(0.234)	(0.603)
Veteran	0.786	0.757	1.238
	(0.189)	(0.149)	(0.307)
Constant	0.0176***	0.00140	2.642
	(0.0194)	(0.00819)	(23.03)
Observations	3010	3813	3135

ACE, adverse childhood experience

* Notes: Columns (1), (2), and (3) report the estimated odds ratios of the relationship between stress related food insecurity and the principal independent variable, the continuous ACE score as well as its interaction with an Appalachian county indicator, and other covariates. Column (1) presents the odds ratios for the 18–44 years old sample. Panel II (Panel III) presents the odds ratios for the 45–64 years old (65 and older) sample. All models include the following control variables: age, squared age, male indicator, an indicator for Appalachian county, education categories, race categories, income categories, a dummy variable for being employed, an indicator for veteran, an indicator for married, an indicator for any children in the household, an indicator for home ownership, and an indicator for renting. The omitted categories are male, not living in an Appalachian county, less than high school education, non-Hispanic white, missing income, unemployed, not a veteran, not married, no children in household, and residing in mobile home or other type of housing. Robust s.e. in parentheses. All analyses weighted by BRFSS sample weights.

Significance:

*** p<0.01,

** p<0.05,

* p<0.1.

See text for definitions of ACE measures.

**Table 3:** Association between food insecurity and any household dysfunction subscale, by age group

	Odds of food insecurity by age group		
Independent variables↓	(1) Respondents aged 18–44	(2) Respondents aged 45–64	(3) Respondents aged ≥ 65
Any household dysfunction in childhood	1.436***	1.544***	1.305*
	(0.155)	(0.168)	(0.193)
Appalachian County	1.141	1.227	1.159
	(0.153)	(0.161)	(0.196)
Female	1.211*	1.571***	1.225
	(0.139)	(0.190)	(0.237)
High school degree or equivalent	0.773	0.717*	0.581***
	(0.131)	(0.124)	(0.106)
Some college	0.911	0.769	0.649**
	(0.161)	(0.141)	(0.130)
College degree or higher	0.607**	0.433***	0.727
	(0.123)	(0.0859)	(0.173)
Black, non-Hispanic	0.705**	0.744**	1.456*
	(0.0990)	(0.104)	(0.303)
Any race, Hispanic	1.181	1.181	1.309
	(0.190)	(0.333)	(0.832)
Other/Multiple race, non-Hispanic	0.750	1.513**	1.583
	(0.158)	(0.315)	(0.478)
Age in years	1.197**	1.345	0.994
	(0.0837)	(0.277)	(0.227)
Squared age	0.998**	0.997*	1.000
	(0.00107)	(0.00187)	(0.00150)
Married or partnered	1.006	0.803*	1.037
	(0.127)	(0.0955)	(0.176)
Currently employed	0.765**	0.573***	1.365
	(0.0884)	(0.0660)	(0.326)
Any children in household	0.982	1.766***	1.967*
	(0.110)	(0.260)	(0.716)
Annual household income <$15,000	1.780***	3.809***	3.405***
	(0.337)	(0.760)	(0.764)
Annual household income $15,000 – 24,999	1.251	2.543***	2.216***
	(0.216)	(0.453)	(0.444)
Annual household income $25,000 – 49,999	0.795	1.046	0.620**
	(0.140)	(0.174)	(0.131)
Annual household income $50,000 – 74,999	0.364***	0.493***	0.226***
	(0.0848)	(0.106)	(0.104)
Annual household income ≥ $75,000	0.241***	0.128***	0.0632***
	(0.0673)	(0.0341)	(0.0415)
Own home	1.277	0.680*	0.835
	(0.267)	(0.155)	(0.251)
Rent	1.811***	0.987	1.861*
	(0.343)	(0.241)	(0.603)
Veteran	0.789	0.812	1.029
	(0.178)	(0.153)	(0.249)
Constant	0.0151***	0.000602	0.700
	(0.0162)	(0.00337)	(6.002)
Observations	3144	4002	3294

ACE, adverse childhood experience

* Notes: Columns (1), (2), and (3) report the estimated odds ratios of the relationship between stress related food insecurity and the principal independent variable, the indicator for any household dysfunction experienced in childhood as well as its interaction with an Appalachian county indicator, and other covariates. Column (1) presents the odds ratios for the 18–44 years old sample. Panel II (Panel III) presents the odds ratios for the 45–64 years old (≥65) sample. All models include the following control variables: age, squared age, gender, an indicator for Appalachian county, education categories, race categories, income categories, a dummy variable for being employed, an indicator for veteran, an indicator for married, an indicator for any children in the household, an indicator for home ownership, and an indicator for renting. The omitted categories are male, not living in an Appalachian county, less than high school education, non-Hispanic white, missing income, unemployed, not a veteran, not married, no children in household, and residing in mobile home or other type of housing. Robust s.e. in parentheses. All analyses weighted by BRFSS sample weights.

Significance:

*** p<0.01,

** p<0.05,

* p<0.1.

See text for definitions of ACE measures.

**Table 4:** Association between food insecurity and any physical/emotional abuse subscale, by age group

	Odds of food insecurity by age group		
Independent variables↓	(1) Respondents aged 18–44	(2) Respondents aged 45–64	(3) Respondents aged ≥65
Any physical and emotional abuse in childhood	1.810***	1.895***	1.562***
	(0.191)	(0.211)	(0.259)
Appalachian County	1.161	1.304**	1.115
	(0.156)	(0.176)	(0.188)
Female	1.224*	1.591***	1.306
	(0.141)	(0.196)	(0.263)
High school degree or equivalent	0.802	0.698**	0.555***
	(0.137)	(0.126)	(0.103)
Some college	0.934	0.788	0.620**
	(0.166)	(0.148)	(0.126)
College degree or higher	0.609**	0.418***	0.705
	(0.124)	(0.0858)	(0.170)
Black, non-Hispanic	0.723**	0.762*	1.519**
	(0.101)	(0.111)	(0.310)
Any race, Hispanic	1.094	1.026	1.372
	(0.179)	(0.285)	(0.884)
Other/Multiple race, non-Hispanic	0.736	1.319	1.586
	(0.153)	(0.290)	(0.478)
Age in years	1.198**	1.287	0.927
	(0.0846)	(0.271)	(0.212)
Squared age	0.998**	0.997	1.000
	(0.00109)	(0.00192)	(0.00151)
Married or partnered	1.000	0.854	1.012
	(0.127)	(0.105)	(0.178)
Currently employed	0.771**	0.588***	1.441
	(0.0904)	(0.0696)	(0.348)
Any children in household	1.017	1.741***	1.774
	(0.115)	(0.263)	(0.667)
Annual household income <$15,000	1.805***	3.655***	3.301***
	(0.348)	(0.760)	(0.776)
Annual household income $15,000 – 24,999	1.256	2.435***	2.101***
	(0.221)	(0.433)	(0.437)
Annual household income $25,000 – 49,999	0.802	1.001	0.551***
	(0.143)	(0.170)	(0.122)
Annual household income $50,000 – 74,999	0.364***	0.465***	0.198***
	(0.0863)	(0.0995)	(0.0932)
Annual household income ≥ $75,000	0.238***	0.121***	0.0578***
	(0.0673)	(0.0320)	(0.0380)
Own home	1.303	0.643*	0.886
	(0.272)	(0.148)	(0.268)
Rent	1.768***	0.932	1.842*
	(0.333)	(0.227)	(0.606)
Veteran	0.723	0.767	1.218
	(0.172)	(0.148)	(0.299)
Constant	0.0147***	0.00204	9.676
	(0.0158)	(0.0116)	(83.06)
Observations	3121	3951	3218

* Notes: Columns (1), (2), and (3) report the estimated odds ratios of the relationship between stress related food insecurity and the principal independent variable, the indicator for any household dysfunction experienced in childhood as well as its interaction with an Appalachian county indicator, and other covariates. Column (1) presents the odds ratios for the 18–44 years old sample. Panel II (Panel III) presents the odds ratios for the 45–64 years old (65 and older) sample. All models include the following control variables: age, squared age, gender, an indicator for Appalachian county, education categories, race categories, income categories, a dummy variable for being employed, an indicator for veteran, an indicator for married, an indicator for any children in the household, an indicator for home ownership, and an indicator for renting. The omitted categories are male, not living in an Appalachian county, less than high school education, non-Hispanic white, missing income, unemployed, not a veteran, not married, no children in household, and residing in mobile home or other type of housing. Robust s.e. in parentheses. All analyses weighted by BRFSS sample weights.

Significance:

*** p<0.01,

** p<0.05,

* p<0.1.

See text for definitions of ACE measures.

**Table 5:** Association between food insecurity and any sexual abuse subscale, by age group

	Odds of food insecurity by age group		
Independent variables↓	(1) Respondents age 18–44	(2) Respondents age 45–64	(3) Respondents ≥ 65
Any sexual abuse in childhood	2.076***	1.652***	1.397
	(0.291)	(0.259)	(0.394)
Appalachian County	1.105	1.354**	1.138
	(0.148)	(0.179)	(0.192)
Female	1.110	1.519***	1.242
	(0.128)	(0.185)	(0.247)
High school degree or equivalent	0.777	0.709**	0.532***
	(0.131)	(0.124)	(0.0944)
Some college	0.884	0.771	0.592***
	(0.155)	(0.142)	(0.116)
College degree or higher	0.592***	0.420***	0.656*
	(0.118)	(0.0843)	(0.156)
Black, non-Hispanic	0.710**	0.804	1.516**
	(0.0996)	(0.116)	(0.310)
Any race, Hispanic	1.089	1.036	1.257
	(0.174)	(0.281)	(0.831)
Other/Multiple race, non-Hispanic	0.717	1.393	1.574
	(0.149)	(0.289)	(0.479)
Age in years	1.178**	1.288	0.966
	(0.0805)	(0.265)	(0.213)
Squared age	0.998**	0.997	1.000
	(0.00105)	(0.00187)	(0.00145)
Married or partnered	0.993	0.846	0.946
	(0.124)	(0.101)	(0.160)
Currently employed	0.771**	0.581***	1.400
	(0.0893)	(0.0670)	(0.331)
Any children in household	1.050	1.814***	1.741
	(0.117)	(0.267)	(0.622)
Annual household income <$15,000	1.657***	3.856***	3.044***
	(0.312)	(0.767)	(0.686)
Annual household income $15,000 – 24,999	1.190	2.478***	1.953***
	(0.204)	(0.432)	(0.393)
Annual household income $25,000 – 49,999	0.766	1.087	0.566***
	(0.133)	(0.180)	(0.119)
Annual household income $50,000 – 74,999	0.340***	0.500***	0.209***
	(0.0795)	(0.107)	(0.0957)
Annual household income ≥ $75,000	0.224***	0.131***	0.0591***
	(0.0627)	(0.0349)	(0.0387)
Own home	1.300	0.639**	0.911
	(0.267)	(0.145)	(0.276)
Rent	1.850***	1.006	1.864*
	(0.343)	(0.243)	(0.607)
Veteran	0.791	0.762	1.078
	(0.181)	(0.148)	(0.259)
Constant	0.0257***	0.00208	2.569
	(0.0266)	(0.0117)	(21.31)
Observations	3149	4013	3294

* Notes: Columns (1), (2), and (3) report the estimated odds ratios of the relationship between stress related food insecurity and the principal independent variable, the indicator for any sexual abuse experienced in childhood as well as its interaction with an Appalachian county indicator, and other covariates. Column (1) presents the odds ratios for the 18–44 years old sample. Panel II (Panel III) presents the odds ratios for the 45–64 years old (65 and older) sample. All models include the following control variables: age, squared age, gender, an indicator for Appalachian county, education categories, race categories, income categories, a dummy variable for being employed, an indicator for veteran, an indicator for married, an indicator for any children in the household, an indicator for home ownership, and an indicator for renting. The omitted categories are male, not living in an Appalachian county, less than high school education, non-Hispanic white, missing income, unemployed, not a veteran, not married, no children in household, and residing in mobile home or other type of housing. Robust s.e. in parentheses. All analyses weighted by BRFSS sample weights.

Statistical significance:

*** p<0.01,

** p<0.05,

* p<0.1.

See text for definitions of ACE measures.
